# Research Progress on Controlled Low-Strength Materials: Metallurgical Waste Slag as Cementitious Materials

**DOI:** 10.3390/ma15030727

**Published:** 2022-01-19

**Authors:** Yiliang Liu, Youpo Su, Guoqiang Xu, Yanhua Chen, Gaoshuai You

**Affiliations:** 1 Deparment of Mining Engineering, North China University of Science and Technology, Tangshan 063210, China; 2 Department of Architectural Engineering, North China Institute of Aerospace Engineering, Langfang 065099, China; yougaoshuai@126.com; 3 College of Civil and Architectural Engineering, North China University of Science and Technology, Tangshan 063210, China; suyoupo@126.com (Y.S.); xgq1973@126.com (G.X.); cyh427@163.com (Y.C.)

**Keywords:** controlled low-strength materials, metallurgical waste slag, flow, compressive strength, supplementary cementitious materials, blast-furnace slag, steel slag, red mud

## Abstract

Increasing global cement and steel consumption means that a significant amount of greenhouse gases and metallurgical wastes are discharged every year. Using metallurgical waste as supplementary cementitious materials (SCMs) shows promise as a strategy for reducing greenhouse gas emissions by reducing cement production. This strategy also contributes to the utilization and management of waste resources. Controlled low-strength materials (CLSMs) are a type of backfill material consisting of industrial by-products that do not meet specification requirements. The preparation of CLSMs using metallurgical waste slag as the auxiliary cementing material instead of cement itself is a key feature of the sustainable development of the construction industry. Therefore, this paper reviews the recent research progress on the use of metallurgical waste residues (including blast furnace slag, steel slag, red mud, and copper slag) as SCMs to partially replace cement, as well as the use of alkali-activated metallurgical waste residues as cementitious materials to completely replace cement for the production of CLSMs. The general background information, mechanical features, and properties of pozzolanic metallurgical slag are introduced, and the relationship and mechanism of metallurgical slag on the performance and mechanical properties of CLSMs are analyzed. The analysis and observations in this article offer a new resource for SCM development, describe a basis for using metallurgical waste slag as a cementitious material for CLSM preparation, and offer a strategy for reducing the environmental problems associated with the treatment of metallurgical waste.

## 1. Introduction

### 1.1. Supplementary Cementitious Materials

**Figure 1:**
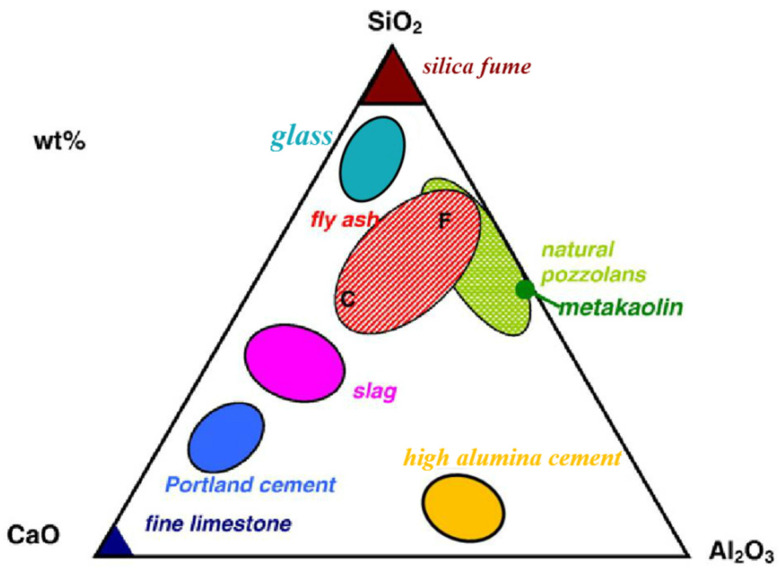
CaO–Al_2_O_3_–SiO_2_ ternary diagram (wt % based) of common cementitious materials [4,5].

The cement industry accounts for about 5–9% of global CO_2_ emissions [1,2]. Therefore, reducing CO_2_ emissions by decreasing cement use via the utilization of supplementary cementitious materials (SCMs) has become an important research field in the building materials industry in recent years. Currently, the literature mainly focuses on SCMs with natural cementation and potential cementation properties, including the most commonly used industrial by-product SCMs, such as coal fly ash and cement kiln embers. These industrial by-products usually do not involve additional calcination processes and can therefore significantly reduce both the CO_2_ emissions per ton and the unit cost of cement-based materials [3]. Pozzolanic materials are the main source of SCMs. A pozzolanic reaction is the reaction of active silicon oxide and alumina with Ca(OH)_2_, which leads to the formation of calcium aluminate hydrate and calcium silicate hydrate (C-S-H). Different SCMs have different chemical elements, mineral compositions, and crystal structures. Consequently, different hydrates form during the hydration process in different SCMs, which affects the strength and durability of concrete. Figure 1 shows a ternary diagram of the CaO-Al_2_O_3_-SiO_2_ chemical composition of commonly used SCMs.

### 1.2. Controlled Low-Strength Materials (CLSMs)

Committee 229 of the American Concrete Institute (ACI) has defined CLSMs as self-compacting cementing materials that are originally used as structural fillers or as backfill instead of compacted soil, that do not need to be rammed or compacted to achieve full strength, and that usually have a much higher load-carrying capacity than compacted soil. These characteristics reduce compaction equipment requirements, labor costs, and the need for related geotechnical testing experiments [6]. CLSMs are easily mixed, have high mobility, and bleed into tight spaces that are hard for compacting equipment to reach. Moreover, CLSMs have a rapid curing speed, reducing the need for a long construction period. In their hardened state, CLSMs are uniform materials of constant density that are less prone to erosion and piping than compacted soil. Therefore, CLSMs are multi-purpose backfilling materials that can be used for trench backfilling, soft soil foundation treatment, urban pipeline cushioning, underground space backfilling (e.g., in mined-out areas and abandoned underground tunnels), and the filling of roadbeds, sidewalks, abutment backs, and erosion control slopes [7]. CLSMs are environmentally friendly, economical, and do not require intensive labor. In the initial stage of pouring, CLSMs behave like soil, due to their high ductility. However, as time passes, CLSMs begin to behave like concrete [8]. Most of the knowledge and literature regarding CLSM applications has bridged the fields of concrete material engineering and geotechnical engineering, but neither of these two areas has received a sufficient level of attention. In addition, CLSMs also have some disadvantages, such as easy bleeding and segregation, a tendency toward volume shrinkage and settlement, and poor durability under freeze-thaw cycles. In CLSM applications, the suitability of a CLSM should be strictly evaluated based on the engineering application and environment, and the performance of CLSMs should be improved by adjusting their material composition.

#### 1.2.1. CLSM Strength

The 28-day unconfined compressive strength of CLSMs is typically less than 8.3 MPa. For backfill projects that are expected to require future excavation, the maximum crushing resistance of the selected CLSM should typically not be higher than 2.1 MPa. CLSMs with compressive strengths of 0.3–0.7 MPa are equivalent to a highly compactable backfill. To improve soft soil and uneven foundations, structural backfill CLSMs used to support building foundations have compressive strength values ranging from 0.7 to 8.3 MPa, depending on the application. The most commonly used CLSMs have compressive strengths of 0.3 to 2.1 MPa. The difference between CLSMs and compacted cement-soil mixtures is that the strength of CLSMs is primarily due to cement-material hydration and because compaction, consolidation, and specific curing are not usually required to achieve the desired strength. Another notable feature of CLSMs is their high fluidity. The fluidity of these materials should be at least 200 mm, and 300 mm or more is required to prevent the clogging up of pumping equipment [9].

#### 1.2.2. CLSM Constituent Materials

Conventional CLSM mixtures are composed of raw materials, such as water, cement, aggregates, and fly ash or other auxiliary cementitious materials. Fly ash has become the most common by-product admixture in CLSMs, due to its glass bead structure and auxiliary gelation effects. To meet material and application requirements, other admixtures are added when necessary. For instance, air outflow or foaming agents can be added to CLSMs to help improve their workability and heat preservation properties, to reduce bleeding, segregation, contraction, and material density, and to control the development of ultimate strength [10,11]. The addition of bentonite can reduce the bleeding rate and permeability of CLSMs. Adding an accelerating agent can reduce the setting and hardening time of CLSMs, which is critical for emergency-repair engineering [12]. Typical CLSM mix ratios are as follows: 80–85% fine aggregate or bulking agents, 10–15% SCM, 5–10% cement, and 250–400 L/m^3^ water. The actual blending proportions may vary between applications, depending on their requirements. Coarse aggregate is usually not added to CLSMs that need to be excavated after hardening. Even if the long-term compressive strength of a CLSM is less than 2.1 MPa, the presence of a coarse aggregate will hinder re-excavation. This is particularly true if manual excavation is required.

CLSMs have low compressive strength values compared with concrete, so some non-standard materials can also be used in CLSM mixtures. Non-standard materials, such as fine aggregate and fly ash that do not meet the specified requirements, can be used as long as the CLSM performance meets the requirements for engineering use. The industrial by-products used in CLSMs can be divided according to their pozzolanic activity into potentially active materials and inert materials. Alkali, salt, or compound activators can be used to stimulate mineral waste residues, fly ash, coal gangue, steel slag, red mud, and other potentially active pozzolanic materials. These activated pozzolanic materials are transformed into cohesive cementitious materials, and they can partially or completely replace the cement in CLSMs. Inert materials, such as mine tailings, stone processing waste, waste glass, the incineration bottom ash of household garbage, and oyster shells are typically used as aggregates to replace natural sand and stone in CLSM preparation.

The selection of raw materials should take into account the applicability, economy, and sustainability of specific applications, as well as the requisite mixture characteristics, such as fluidity, hardening strength, volume stability, and density [13]. The most recent studies on CLSMs focus on the effects of using industrial sector by-products or waste, as the main CLSM components, on the working and mechanical properties of the resulting CLSM mixtures. Due to the incorporation of by-products, some researchers have also studied the environmental acceptability of CLSMs.

#### 1.2.3. Commonly Used SCMs in CLSMs

Due to the low strength and cost requirements of CLSMs, many industrial by-products are used as SCMs to replace some or all of the cement in the production of CLSMs and mine-filling materials. These SCMs can be divided into the following categories: industrial incineration by-products, metallurgical industrial by-products, cement kiln ash, and alkali-activated cementitious materials. Among the most commonly used incineration industrial by-products, coal fly ash is the most popular SCM, due to its many advantages, which include a glass bead morphological effect, pozzolanic activity, and the microaggregate effect of the very fine beads. Coal fly ash appears in nearly 38% of the literature related to CLSMs [14,15]. Moreover, because incineration ash usually exhibits pozzolanic activity, other types of incineration ash have also been applied as SCMs for CLSM production. These include incineration sludge ash, such as waste paper sludge ash (WPSA) [16], incineration sludge ash [17], and mixed incineration sludge ash [18]; garbage incineration ash, such as domestic waste incinerator bottom ash [19] and industrial waste incineration bottom ash [20]; and biomass incineration ash, such as wood ash [21], corn straw ash [22,23], rice straw ash [24] and bagasse ash [25]. Among the metallurgical industrial by-products, blast-furnace slag is often used as an SCM for CLSM production, due to its high hydration activity. In addition, steel slag [26,27], ferrochrome slag [28], red mud [29], copper slag [30], and lithium slag [31] are often used in CLSM production.

The selection and use of SCMs are not limited to a single SCM or a single category of SCMs. Instead, several SCMs or multiple types of SCMs may be used simultaneously. For instance, fly ash and blast-furnace slag may be combined. Lin et al. [32] prepared a CLSM by mixing water-quenched blast-furnace slag, cement and CFB fly ash at a ratio of 7:2:1. This mixture was used as a cementitious material and combined with fine aggregates. Zhou et al. [33] used fly ash, quicklime, and blast-furnace slag as SCMs to partially replace cement, as well as aeolian sand as a fine aggregate, to prepare low-strength filling materials. This was used for filling goafs near a desert. Chompoorat et al. [34] prepared a CLSM without cement by mixing fly ash, steel slag, sodium hydroxide, and water with bottom ash (BA) aggregate. The addition of steel slag improved the fluidity, setting time, and compressive strength of their CLSM sample.

### 1.3. Present Situation of Metallurgical Waste Residue Resource Utilization

Metallurgical residues are the solid waste produced by the metallurgy industry. With increasingly rapid global urbanization and industrialization, the continuously increasing demand for metal-based products has inevitably led to the production of a large amount of metallurgical waste residues. Additionally, increasingly strict laws and regulations, taxes, and a shortage of landfill space mean that the cost of treating metallurgical waste residues is increasing every year. Simple landfill disposal may allow some of the toxic and harmful components in metallurgical waste slag to threaten the ecological environment [35]. Therefore, enhancing the resource utilization and effective management of metallurgical slag is an urgent goal that must be met to improve the recovery rates of these materials. Many studies have explored the utilization of metallurgical waste slag as a resource, and some achievements have been successfully reported. These include extracting valuable metallic elements from waste slag, returning waste slag to blast furnaces for re-sintering, and using slag to repair and improve soil environments, prepare cement, and prepare geopolymers [36].

There are three main categories of metallurgical waste slag: ferrous metal-smelting slag, non-ferrous metal-smelting slag, and red mud. Ferrous metal-smelting slag refers to the slag from smelting furnaces, ferroalloy slag, steel slag, and the small amount of iron oxide slag produced in steel-rolling processes. Non-ferrous metal-smelting slag includes copper slag, lead slag, zinc slag, and nickel slag. Red mud is the waste residue of alumina extraction from bauxite. The preparation of building materials is one of the main strategies for achieving the large-scale utilization of metallurgical waste slag. However, the properties of slag vary significantly, due to the different raw materials and metallurgical processes that generate slag. Based on the different characteristics of metallurgical waste slag, scholars have carried out a wide range of treatment and utilization research. Pribulova et al. [37] produced road concrete using sodium silicate and blast-furnace slag particles. Giannopoulou et al. [38] reported a geological convergence reaction using waste mining products. The red mud in their work was produced by extracting alumina from the slag generated by a nickel-iron production process. The inorganic polymer materials prepared by this geopolymerization reaction formed an amorphous aluminosilicate gel phase and were well-combined with undissolved solid raw material particles. Thus, excellent physical and mechanical properties were exhibited. Gholizadeh et al. [39] studied Fe-free metal slag as a cementing material in a mixture. They treated copper slag, lead slag, and synthetic ferric silicate slag via thermal metallurgy to reclaim precious metals and to remove heavy toxic components and sulfate, then quickly cooled the slag in water. Their experimental results showed that the three investigated metallurgical slags exhibited average pozzolanic activity. These slags can also be chemically modified by adding calcium and aluminum additives in the heat-treatment process, and the reactivity of the slags can be improved by rapid cooling. Y. Zhang et al. [40] reported the solidification ability of precious metallurgical slags from tailings high in arsenic, for use as cementing materials.

The production of Portland cement is an energy-intensive industry. Approximately 400 MJ of thermal energy is required per ton of cement. This thermal energy accounts for the grinding of raw materials, raw-material calcination at 1500 °C, and the milling of lime and caster. However, rapidly cooled iron waste has pozzolanic attributes and can be used as a cementing material after undergoing a grinding process, without the need for calcination. Grinding metallurgical waste slag saves a significant amount of energy. For example, the energy required to grind granular blast-furnace slag is approximately 10% of the total energy required to produce Portland cement [41]. Therefore, utilizing metallurgical waste as an SCM is an ideal strategy for lowering the usage and cost of fuel for Portland cement production.

A great deal of research has been conducted into the preparation of CLSMs from various metallurgical waste residues, and outstanding results have been achieved. CLSMs have been prepared that reduce the consumption of cement, and some CLSMs demonstrate better working performance or mechanical properties than single cementitious materials in cement. In addition, metallurgical waste slag can be used in place of cement. This results in the effective disposal of bulk solid waste and improves the economic benefits of these construction materials. In view of the wide adaptability of CLSMs to raw materials, metallurgical waste materials other than blast-furnace slag should be thoroughly developed as substitute auxiliary cementing materials in the production of CLSMs. This strategy is expected to offer an emerging strategy for processing metallurgical waste. Moreover, this strategy will reduce the demand for energy and raw materials in construction engineering. The selected materials reviewed in this paper include academic papers, industry standards, online articles, and research reports. In total, 153 references were used for this study.

## 2. Production of CLSMs from Metallurgical Waste SCMs

### 2.1. Blast-Furnace Slag

#### 2.1.1. Overview of Blast-Furnace Slag

Blast-furnace slag is a silicate melt formed by the reaction of substances, such as SiO_2_ and Al_2_O_3_, contained in Fe ore with CaO and MgO in flux during raw iron melting. Air-cooled slag is obtained from a melt as a result of air-cooling treatment and is generally used as a slag aggregate. Granulated slag is formed after granulation treatment and can be used as a cement mixture or light aggregate. Water-quenched slag is obtained after a water-quenching treatment; this slag is highly porous. Finally, irregular and glassy amorphous materials can be obtained through the formation of blast-furnace slag powder with pozzolanic activity, after drying and grinding [42]. Slag water-quenching processes are rapid to complete, and some of the unreleased heat energy of these processes is converted into chemical energy and stored as chemical bonds in the slag. Consequently, water-quenched blast-furnace slag structures are unstable materials with high energy. Water-quenched blast-furnace slag, therefore, offers a high potential cementing activity. The word “slag” in this paper usually refers to water-quenched slag. Water-quenched slag is primarily discharged with granulation and must be ground prior to further use. The glass content of ground blast-furnace slag powder accounts for 80–90% of the total slag weight, meaning that slag powder shows good activity under the action of cement clinker or other activators [43]. In 2017, the global production of molten iron in blast furnaces was about 1.18 billion tons. Assuming that the ratio of blast-furnace slag to molten iron is 320 kg/t, approximately 380 million tons of blast-furnace slag is produced annually [44]. Therefore, if blast-furnace slag is not appropriately handled on a large scale, this will lead to significant environmental pollution and a waste of resources. Due to the increasing global emphasis on climate change mitigation and carbon emission reduction, the challenges of slag treatment and utilization have attracted an increasing amount of attention around the world. Consequently, an increasing amount of research into slag has been published. At present, slag is mainly used in conjunction with cementitious materials. The utilization of slag to partially replace cement in the production of cement-based materials is a widely employed method for slag resource utilization, and it is possible to replace 10–90% of Portland cement. Ground-granulated blast-furnace slag (GGBFS) is widely used and is a valuable SCM. The research depth and application degree of GGBFS are equivalent to those of fly ash. At present, more than 90% of blast-furnace slag (BFS) is used as a cement clinker substitute or as a cementitious component in cement-based mixtures [45,46]. In Poland, 100% of BFS is already used to produce cement [47].

#### 2.1.2. Physical and Chemical Properties of Blast-Furnace Slag

Granulated blast-furnace slag (GBFS) is produced due to the rapid quenching of molten BFS with a high-pressure water jet. GBFS consists of glassy granular particles with typical granule dimensions of ≤ 5 mm, similar to that of sand. The particle shape of GBFS changes from a sub-angle to a sub-circle. Generally, when used as an SCM, GBFS is ground down to obtain GGBFS. The surfaces of GGBFS particles are smooth and dense, forming a smooth-sliding surface. GGBFS particles therefore only absorb a small amount of water. When GGBFS is used to partially replace cement, the resulting mixture is more workable. Keeping the proportion of slag slightly lower than that of cement is conducive to the fluidity of the mixture. Furthermore, for the same performance requirements, water consumption can be reduced by up to 10% by using GGBFS [48]. The hydration rate of slag is lower than that of cement, and the partial replacement of cement with slag reduces the water consumption in early hydration. Therefore, concrete containing GGBFS maintains its workability for extended periods and has good durable workability. GGBFS particles are usually finer than cement particles, so GGBFS has a larger specific surface area compared with cement. GGBFS powder particles with good dispersion can be used to fill the gaps between cement particles, replacing the filling water and improving the free water in these mixtures. This enhances fluidity. However, these powder particles also consume water due to surface wetting and adsorption. If the water consumption that is necessary to fill the gaps between cement particles is greater than the water consumption required by powder wetting, the fluidity of the mixture will be improved by using GGBFS. Otherwise, the water consumption of the mixture will increase. Therefore, it should be noted that higher GGBFS fineness does not necessarily lead to better properties and that not all fine slags are conducive to fluidity. One drawback of slag-modified concrete is that at temperatures below 23 °C, the setting time of the slag-modified concrete is significantly delayed, compared with that of slag-free concrete. This negatively impacts concrete pouring in winter, especially in terms of emergency repair projects.

The initial ore type, dosage, metallurgical process, processing method, storage, and weathering times of slags affect their properties. Slag is mainly composed of CaO, SiO_2_, Al_2_O_3_, MgO, and smaller amounts of other oxides. GGBFS is one of the most widely studied and effective cement replacement materials currently used in concrete manufacturing [49]. Table 1 shows the following chemical ingredients of several representative slags from countries and regions. It varies greatly, relying on the region of origin, with CaO content ranging from 35.66% to 51.65%, SiO_2_ content ranging from 25.56% to 38.7%, Al_2_O_3_ content ranging from 7.7% to 17.30%, and MgO content ranging from 2.43% to 10.3% [50,51,52,53,54,55,56,57,58]. However, the proportions of the CaO, SiO_2_, Al_2_O_3_, and MgO components of slag resemble the proportions found in Portland clinker [59]. The main chemical components of Portland cement clinker are CaO, SiO_2_, Al_2_O_3_, and Fe_2_O_3_. These four oxides account for more than 95% of the total mass of Portland cement clinker. CaO is 62–67%, SiO_2_ is 20–24%, Al_2_O_3_ is 4–7%, and Fe_2_O_3_ is 5–6% of the total Portland cement clinker mass [60]. Compared with Portland cement clinker, slag has less CaO and Fe_2_O_3_ but a significantly higher amount of SiO_2_, Al_2_O_3_, and MgO. Compared with typical pulverized coal, slag has less SiO_2_ and Al_2_O_3_ content but significantly higher CaO and MgO content. The chemical composition of slag directly affects its reactivity, meaning that the activities of slags from different sources will differ to some extent. Therefore, slag can be classified according to its activity indicator.

In 1862, the German businessman Emil Langen discovered the potential hydraulicity of GGBFS. Since then, slag has been widely used in cement mixtures. In 1909, Germany introduced the first standard for using GGBFS in cement manufacturing. Due to the in-depth understanding of slag, slag application technology has matured and some countries and regions have therefore promulgated different versions of standards for the use of slag in cement materials. Table 2 summarizes some typical slag standards. In the Chinese standard GB/T 18046 [61], slag is divided into three grades (S109, S95, and S75) according to its activity index. Chinese standard GB/T 18046 also limits the content of harmful chemicals, such as sulfur trioxide and chloride ions in slag. The Japanese standard JIS A 6206 [62] also classifies slag according to activity index and specific surface area, and this standard includes four grades: 8000, 6000, 4000, and 3000. Compared with the Chinese standard, the Japanese standard has a higher specific surface area requirement for slag, with the same 28-day activity index. Korea’s standard for ore powder is KS F 2563 [63]. This standard divides ore powder into three grades (Class 1, Class 2, and Class 3), based on the activity index. The requirements of the Korean standard are slightly higher than those of the Japanese standard. The EU standard EN 15167-1:2006 [64] does not classify slag, only requiring a specific surface area of ≥275 m^2^/kg and a 28-day activity index ≥ 70%. The standard for slag in the United States is the ASTM C989/C989M-18a standard, published in 2018 [65], and this standard does not have density or specific surface-area requirements. Instead, mineral powders are divided into three grades, based on their activity index. In the above period of validity, Grade 120 is ≥115%, Grade 100 is ≥95%, and Grade 80 is ≥75%. It should be noted that the Australian standard AS 3582.2:2016 [66] does not adopt the specific surface area and activity index requirements commonly used internationally. Instead, AS 3582.2:2016 emphasizes the chemical composition of slag. The Australian standard specifies that the composition of slag should be as follows: iron oxide ≤1.5%, magnesium oxide ≤15%, and alumina ≤18%. In contrast, the content of these three oxides is not specified by the Chinese national standard. The knowledge and implementation of local standards and relevant testing methods are necessary to fully utilize slag resources.

#### 2.1.3. Hydration Activity of Blast-Furnace Slag

The fluidizing effect of slag is influenced by its properties, both chemical and mechanical. These include its glazing capacity, chemical composition, mineral composition, and fineness. Based on the national and regional criteria described in Section 2.1.2 and Table 2, activity is an important index for evaluating the effectiveness of pulverized composite materials, and most standards generally classify slag according to its activity index and specific surface area. As with all cementing materials, the specific surface area is an important parameter in determining the hydration activity, early strength development, and water demand of concrete. As a cementing material, slag usually has a high specific surface area. Increasing the specific surface area of slag increases its contact area with water, allowing hydration reactions to be carried out across a larger surface area. Thus, slag responsiveness is enhanced for better compressive strength development. Generally, a specific surface area range of 550–650 kg/m^2^ is acceptable [67]. However, excessively fine slag particle sizes will increase the GGBFS grinding cost and the required amount of mixing water. Moreover, excessively fine particles have adverse effects on shrinkage, cracking, setting time, and other aspects of cementing materials. In addition, the glass phase content of slag is critical to its reactivity. A greater crystal content in slag usually reduces its hydraulic activity. Generally, the glass phase content of slag should exceed 90% to obtain satisfactory performance [68]. The Chinese standard GB/T 18046 [61] clearly requires that the glass phase content of S105 and S95 slag should be ≥85%.

The hydraulic activity of slag is enhanced by CaO, Al_2_O_3_, and MgO content but reduced with increasing SiO_2_ content. The EU standard EN 15167-1:2006 [64] requires that (CaO + MgO + Al_2_O_3_) ≥2/3 and (Cao + MgO)/SiO_2_ ≥ 1. The Japanese standard JIS A 6206:2013 [62] requires that (CaO + MgO + Al_2_O_3_)/SiO_2_ ≥ 1.6, while the Chinese standard GB/T 203-2008 [69] requires that (CaO + MgO + Al_2_O_3_)/(SiO_2_ + MnO + TiO_2_) ≥ 1.2. It should be noted that CaO content exceeding this limitation makes granulation difficult, leading to a reduction in glass phase content [70]. The activity of MgO is lower than that of CaO. When CaO content is insufficient, a higher MgO concentration can be used to compensate for alkalinity. Changing the MgO content of slag by about 8–10% reduces the impact on resistance evolution, but high MgO content may have the reverse effect. The Japanese standard JIS A 6206:2013 [62] requires that MgO content ≤ 10% and the Australian standard AS 3582.2:2016 [66] requires that MgO content ≤ 15%. Due to the complexity of the factors affecting the hydration reaction of slag, the activity of slag cannot be accurately evaluated by merely analyzing its glass phase content or chemical composition. In most specifications and studies, the slag activity index (SAI) is used as the fundamental standard to evaluate gelation.

When slag is ground down into particles smaller than 45 μm, self-gelation occurs, but the hydration rate is slow. The short-term compressive strength of slag concrete is lower than that of Portland cement (OPC) concrete, but the reactivity of different slags may significantly vary under the same conditions. Although the slower hydration rate of slag is unfavorable for early hardening (e.g., 3 or 7 days), it is favorable for the development of 28-day strength and longer-term strength. Compared with OPC concrete, concrete that contains GGBFS can still develop strength at a higher rate in the later hardening stage. Even several years after casting, large quantities of non-reacted waste slag particles are still present in the slag cement slurry. The presence of these non-reacted slag particles in the alkaline solution environment of cement contributes to the ability of these materials to self-heal cracks [71].

The mechanism of GGBFS hydration is different from that of cement, silica fume, fly ash, and other pozzolanic materials. GGBFS has self-gelling properties, while fly ash only has self-gelling properties when its CaO content is high. The hydration of GGBFS relies on the destruction and melting of its glazing phase, caused by the release of hydroxyl ions during slag hydration. GGBFS hydration consumes Ca(OH)_2_, resulting in the generation of additional C-S-H gel. When cement reacts with water, C-S-H forms near the cement particles. Ca(OH)_2_ migrates and precipitates into discrete crystals in pore solutions, surrounded by large pores. When slag is mixed with Portland cement, rapid fluidization of the slag occurs due to the existence of this Ca(OH)_2_ [72]. In addition, GGBFS reacts with the Ca(OH)_2_ to create a finely distributed gel that can fill larger pores [73]. Therefore, hardened slag cement slurry contains fewer hydroxide crystals and fewer large, filled capillary pores. In general, the hydration of GGBFS with cement is a two-stage reaction. The initial stages of hydration mainly consist of a reaction with alkali metal hydroxides, and the later stage is mainly an experimental reaction with Ca(OH)_2_ [43]. The final hydration product after slag activation is similar to the cement C-S-H, but the Ca/Si ratio of the slag hydration product is lower. Therefore, the activation rate and strength of slag significantly differ compared with those of ordinary Portland cement [49].

#### 2.1.4. Properties and Mechanism of Slag CLSM

- Mechanical properties and mechanism

**Figure 2:**
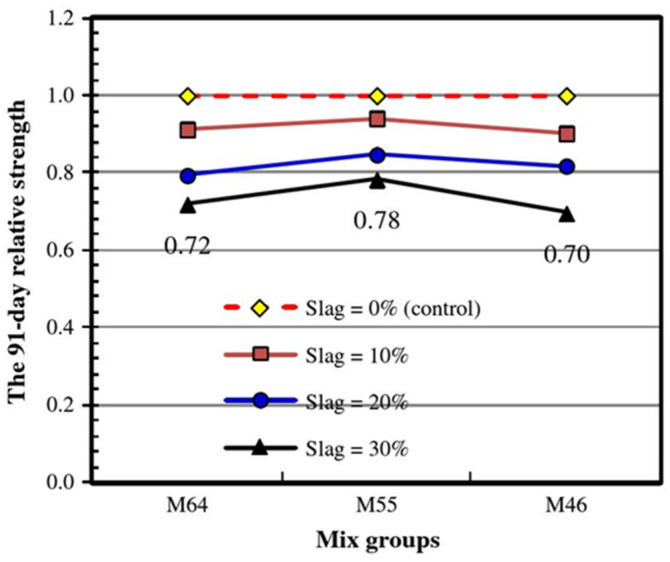
The 91-day relative compressive strengths of slag-containing CLSMs, compared to a slag-free control mixture [74].

In view of the hydration properties of slag, the preparation of CLSMs when using slag as an SCM has been extensively investigated in the literature. Generally, as the proportion of slag replacing cement increases, the flow performance of fresh CLSM mixtures increases and their compressive strength decreases. In the production of CLSMs with slag, cement plays a leading role in the development of the CLSM’s compressive strength. This is similar to CLSM production with other SCMs. Using more cement results in greater compressive strength. Generally, the hydration performance of SCMs is lower than that of cement, so the compressive strength of CLSMs decreases as the proportion of SCMs replacing cement increases. The results of Sheen et al. [74] proved this trend. As shown in Figure 2, they defined the relative compressive strength as the strength ratio of slag-containing and slag-free specimens after 91 days of curing. M64, M55, and M46 represent the different proportions of sand and soil in the aggregate. Figure 2 clearly shows that the incorporation of slag lowered the strength of the CLSM. When the proportion of slag replacing cement was 30%, the 91-day strength reached 72%, 78%, and 70% of that of the control samples, and the strength ratio was always lower than 1. This is because slag has a lower CaO/SiO_2_ ratio than cement and is inferior to cement in terms of strength development. This is especially true for high water–binder ratios, where “binder” refers to both the cement and the slag. It should be noted that CLSMs have a higher water–binder ratio than concrete. Although the water–binder ratio remained constant in the work of Sheen et al., this ratio increased due to reduction and the lack of cement in the binder. The reduced cement content did not provide enough Ca(OH)_2_ for the reaction with the slag. Hence the higher water–cement ratio was another reason for the reported decline in strength.

Raghavendra and Udayashankar [75] also used cement and slag as cementing materials. In their work, the ratio of cementing material to sand was 1:1.5 and the GGBS/cement ratios were 3.5, 2.8, 2.0, and 1.75. The 28-day compressive strengths of their CLSMs under all mixing ratios were greater than 11.87 MPa. The high strengths of their slag-containing CLSMs indicated that these CLSMs were not suitable for use in projects requiring re-excavation. However, due to their high strengths, they could be developed as a structural backfill material for applications in which re-excavation would not be required. However, the strength of slag-containing CLSMs, as reported in other literature, meets the re-excavation requirement of having a compressive strength lower than 8.3 MPa. D.F. Lin et al. [76] used cement, slag, and fly ash to produce composite cementing materials with dosages of 90 kg/m^3^, 55 kg/m^3^, and 55 kg/m^3^. The total cementing material dosage was 200 kg/m^3^, the ratio of cementing materials to aggregate was 1:8, and the 28-day strength of their resulting CLSM was 8.25 MPa. Sheen et al. [74] found that the 91-day compressive strengths of CLSMs with different mixing ratios were lower than 0.9 MPa. The total binder content (cement and slag) in Sheen’s experiment was only 95 kg/m^3^ and the ratios of cementing material to sand were 1:11.5 to 1:7, far lower than the 1:1.5 ratio found in Raghavendra and Udayashankar’s work [75]. This indicates that the cementing material dosage is a vital parameter that plays a significant role in the development of CLSM’s compressive strength. Therefore, based on whether or not a project requires excavation, CLSM strength can be modified by adjusting the dosage of cementing materials.

In summary, the main factors affecting CLSM strength are the amount of binding material and the proportion of highly active components in the binding material (such as cement). When the total amount of binding material is fixed, a higher proportion of highly active material is more conducive to increasing the strength of the mixture. Weng et al. [77] also demonstrated this trend. In addition, both the water–binder ratio and the water–solid ratio affect the strength development of CLSMs. Using a large amount of data, Du et al. [78] proposed a CLSM strength prediction model using the water–cement ratio, aggregate type, SCM type, and SCM dosage as variables.

The experimental characterization of the mechanical properties of CLSMs is important for their development. Sheen et al. [74] indirectly characterized the compressive strengths of CLSMs by using an ultrasonic pulse velocity method. The pulse velocity of CLSMs gradually increases with the development of their strength, and increasing the slag replacement rate results in a significant decrease in the pulse velocity. Lin et al. [76] used dynamic cone penetration (DCP) to determine the weight-bearing capacity of CLSM foundations and road bases. Compared with the in-situ California bearing ratio (CBR) method, DCP does not require test pits to be dug or soil samples to be collected, and it has the benefits of simple calculations and low equipment requirements. Raghavendra and Udayashankar [75] established a phenomenological model for CLSM production with slag. By inputting the desired flow and strength indices into the model, the water content required for the corresponding fluidity and the binder/water ratio required for the target strength can be obtained. Verification shows that this prediction model is within the precision range required for engineering applications. Moreover, this phenomenological model improves engineering decision-making speed by minimizing the required number of CLSM laboratory tests. Udayashankar and Raghavendra [79] also established a phenomenological model for CLSM preparation, using fly ash and slag as the SCMs instead of cement to facilitate the mix ratio design.

In the past, hydration products and the strength development mechanisms of cement-based mixtures were investigated by microscopic methods such as X-ray diffraction (XRD) and scanning electron microscopy, coupled with energy-dispersive X-ray spectroscopy (SEM-EDS). However, due to the low strength requirements of CSLMs, the cost of binding materials is much lower than that of structural concrete. Hence, the binding material content in CLSMs is low, resulting in a low number of hydration products, and it is not convenient to observe these hydration products by microscopic means. Therefore, no relevant research literature on the correlation between the microstructure and strength development of slag-containing CLSMs has been retrieved.

- Performance of fresh mixtures

The amount of mixing water is the most important factor in determining the flow performance of CLSMs. When the proportion of other materials in a mixture is fixed, the fluidity of CLSMs increases with increasing water consumption. However, increasing the water consumption may also induce bleeding, segregation, and strength reduction [80]. A tendency toward enhanced workability with high slag concentrations has been observed and reported in numerous publications. When the water–binder ratio is fixed, the CLSM flow index increases as the proportion of GGBS replacing cement increases. Sheen et al. [74] showed that when the proportion of slag replacing cement increases from 0 to 30%, the slump spread value increases by 46%. The increased fluidity of GGBFS can be explained as follows: (1) the smooth surface of GGBFS reduces the flow friction resistance; (2) the water absorption rate of GGBFS is lower than that of cement, meaning that when GGBFS partially replaces cement, water absorption is reduced; (3) the hydration rate of GGBFS is also lower than that of cement, meaning that its consumes less water and hinders the formation of ettringite in the early stage of strength development. Tang and Cheng [81] further confirmed this explanation. They evaluated the influence of each selected factor on material performance through the signal-to-noise ratio and further analyzed the influence of each factor on indicators such as liquidity and strength with an ANOVA statistical method. They found that the proportion of cement to water is the most significant factor affecting the fluidity objective level of these mixtures (78%), followed by the slag content (18.71%).

The setting time of CLSMs is prolonged and their ball drop value increases with increasing slag replacement level. This is because slag has pozzolanic properties, a grinding particle size smaller than 45 μm, weak hydraulic characteristics, and a hydration speed slower than that of cement. However, the research of Lin et al. [76] states that even if the use of slag prolongs the setting time of CLSMs, the average elastic modulus of a CLSM can reach 400 MPa after only one day of curing. This is higher than the 386 MPa elastic modulus of conventional backfill. Their results showed that after one day, the poured CLSM reached the mechanical strength of conventionally compressed earth and could support infrastructure loads and vehicle transportation. Therefore, CLSM usage is suitable for these applications and can minimize road closure time.

This section only describes studies using slag as the sole SCM to partially replace cement. Other studies have been reported that aim to enhance the performance of slag SCMs and to improve the utilization of industrial by-products by using two or more industrial by-products together, functioning as SCMs to replace cement. For example, Arivusudar and Babu [82] used GGBFS and fly ash as SCMs to replace cement in order to reduce cement utilization to the greatest extent. Lachemi et al. [83] reported the utilization potentiality of cement kiln dust (CKD) and slag, to completely eliminate cement in the production of CLSMs. They reported that slag had a significant promotion effect on the compressive strength of CKD-based CLSM mixtures with a constant CKD dosage. Raghavendra et al. [84] used cement, slag powder, and waste gypsum wallboard as a new ternary mixed binder, with stone powder as an aggregate to completely replace natural sand in a CLSM. They reported that the addition of GGBFS effectively overcame the harmful effects of the sulfate from the waste gypsum wallboard. Although GGBFS has good hydration properties, its gelation performance is weak. As an SCM, GGBFS usually needs to be combined with cement because the alkaline environment of cement hydration promotes the pozzolanic properties of the slag. If slag completely replaces cement, other alkaline materials are usually required to stimulate slag activity. Research on the preparation of cementless CLSMs by alkali-activated slag will be introduced in detail in the following sections.

### 2.2. Steel Slag

#### 2.2.1. Overview of Steel Slag

Steel slag is a by-product produced in steelmaking and refining processes, and steel slag emissions account for about 15% of the total emissions of crude steel output. According to data released by the World Steel Association, global crude steel output in 2020 was 1.878 billion tons. Of this, China’s crude steel output was 1.065 billion tons, ranking first around the world and accounting for 56.7% of the total global crude steel output [85]. Advanced research has shown that steel slag can be used as an aggregate, due to its excellent engineering properties and good compactness. Steel slag has two inactive substances, free CaO and free MgO. These substances nearly double in size, due to hydration in the later hardening stage of cementing materials, and their expansion over time leads to surface damage or structural damage in hardened steel slag concrete [86]. Therefore, steel slag needs to be aged to prevent volume expansion before it can be used as an aggregate.

#### 2.2.2. Physical and Chemical Properties of Steel Slag

Due to the complexity and diversification of steel slag-smelting processes and treatment processes, there are many types of steel slag and significant differences in slag condition. The overall components of all steel slag specimens are similar, but the content of these chemical constituents can significantly fluctuate. The components of steel slag mainly include CaO, SiO_2_, Al_2_O_3_, Fe_2_O_3_, and MgO, a similar composition to that of cement clinker. However, the content of CaO and SiO_2_ in steel slag is lower than that of Portland cement, which means that the content of calcium silicate in steel slag is low [87]. This calcium content mainly includes dicalcium silicate (C_2_S), tricalcium silicate (C_3_S), a small amount of free calcium oxide (f-CaO), tetracalcium ferroaluminate (C_4_AF), and some metal oxides [88]. C_2_S and C_3_S in steel slag are somewhat active and can be hydrated to form C-S-H gel and calcium hydroxide (CH) crystals, similar to the hydration process of Portland cement [89]. However, silicate minerals and ferroaluminate minerals with relatively high activity in steel slag account for 40–70% of steel slag. In steel slag production, high-temperature melting leads to a more compact C_3_S structure. Consequently, the cementing performance of steel slag is far lower than that of cement clinker, and the use of steel slag results in weak Portland cement [41]. The lower amount of C_3_S and low hydration activity in steel slag mean that a longer time is required for Ca^2+^ ions in the reaction system to reach saturation, to combine with OH^−^ ions after saturation is achieved, and to precipitate Ca(OH)_2_ crystals. Therefore, the reaction rate of steel slag in the early stage is slow and the initial setting time is long. To overcome steel slag’s disadvantages of low activity and insufficient early strength, steel slag can potentially be stimulated by proper activation. The most commonly used activation methods include physical activation, chemical activation, and thermodynamic activation. For example, Wang et al. [90] reported that an ultrafine steel slag prepared by a mechanical grinding method had significantly improved early and medium-term activity. Wang et al. [91] believe that the presence of OH^−^ is conducive to the decomposition of the glassy phase in steel slag and that basic conditions stimulate the early activity of silicates and aluminates. Muhmood et al. [92] remelted and water-quenched the steel slag of an electric arc furnace to provide a slag with both gelling and pozzolanic properties.

#### 2.2.3. Properties and Mechanism of Steel Slag CLSMs

Steel slag has been used as geotechnical backfill material in applications with relatively low strength requirements, such as dock walls, breakwaters, and pipe trenches. Kang et al. [93] used the steel slag powder of an alkaline oxygen furnace as a cementing material to stabilize dredged marine soil. This cementing material acted as a low-strength cement-free backfill material. The fluidity of this backfill material was mainly controlled by the water capacity in the dredged earth, and fluidity increased with the increasing initial water content. In addition, with increasing slag usage, fluidity tended to decrease. Kang et al. [93] proposed that the undrained shear strength of samples in the early curing stage (0.5–10 h) of their mixture should be used to characterize the fluidity of the mixture. With increasing undrained shear strength, the fluidity of their mixture decreased, especially in the case of a sample with a fluidity greater than 100 mm. The compressive strength of backfill materials containing steel slag increased with increasing steel slag content and decreased with increasing initial water content in the dredged soil. This study found that compressive strength did not significantly develop within 0–2 h of curing. In contrast, Seng and Tanaka [94] used cement to stabilize dredged soil, to produce a high level of strength after only 30 min, indicating that steel slag-based mixtures have a longer setting time compared with cement-based mixtures. After curing for 5 h, the strength of Kang’s steel slag-based mixture rapidly developed, the hydration reaction of C_2_S in the steel slag occurred, and the pozzolanic reaction of Ca(OH)_2_ with Si and Al in the dredged soil also took place. In the absence of cement, a higher slag dosage led to a greater mixture strength. A layer of dense amorphous C-S-H gel was deposited on the surface of the steel slag, indicating that the steel slag was an active material. Ettringite and single sulfur calcium sulfate aluminate (AFm) were also found by scanning electron microscopy.

**Figure 3:**
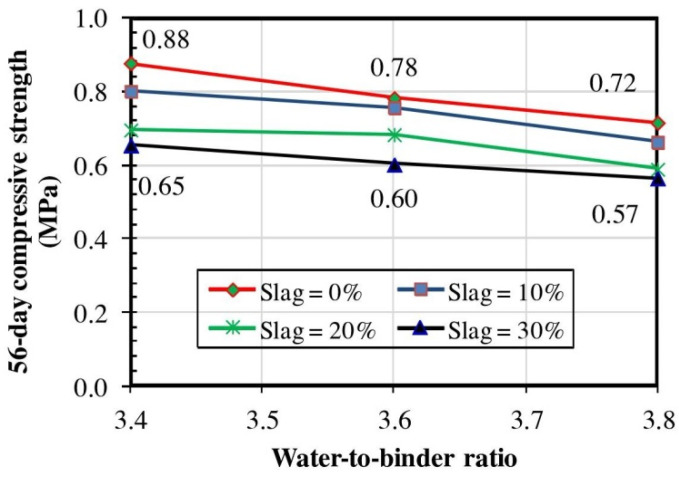
The 56-day compressive strength of CLSMs produced using SSRS and cement with respect to the water–binder ratio [98].

Stainless steel reduction slags (SSRS) are discharged from the reduced state of the basic stainless steel refining process, which is also known as a secondary steelmaking operation. The metal oxides contained in SSRS are similar to those of GGBFS that are produced by iron smelting, but SSRS also contains a few toxic components [95]. Sheen et al. [96] and Le and Nguyen [97] conducted comprehensive studies on the preparation of CLSMs using SSRS instead of cement. Generally, as the proportion of SSRS replacing cement increases, the CLSM performance slightly increases, the setting time increases, the ultrasonic pulse speed decreases, and the compressive strength decreases. Fluidity is mainly controlled by the water capacity and water–binder (W/B) ratio. Increasing water content may lead to high fluidity. The influence of SSRS incorporation on fluidity, as reported by Sheen et al. and Le and Nguyen, is inconsistent with Kang’s conclusion [93], due to the different types of steel slag used and the fact that SSRS are milled and have a more regular surface area. As a potential cementing material, SRSS can delay the formation of ettringite during early hydration, which results in high workability [98]. In terms of setting time, because the hydration rate of SSRS is slower than that of cement, the cement content decreases with an increasing SSRS substitution rate, leading to a longer CLSM setting time. This is especially true under high water–binder ratio conditions [97]. A very high W/B ratio in CLSM compounds leads to an even greater separation of the cementing material particles, resulting in reduced interaction between the cementing materials in the initial hydration stage. Falling ball weight tests further demonstrate the influence of a high cement replacement rate and high W/B ratio on the elongation of setting time. In terms of strength, the W/B ratio, cement content, and aggregate characteristics are generally considered to be the main influencing factors. Increasing the W/B ratio may lead to the generation of high pore volumes in the cement matrix and significant strength reduction. Due to the slow hydration characteristics of SSRS and the inferiority of SSRS compared to cement in terms of strength, the compressive strength decreases as the proportion of SSRS replacing cement increases, especially in the early stage of strength development (Figure 3). Ultrasonic pulse velocity tests can be used as an indication of CLSM strength. Higher pulse velocities in materials are caused by lower porosity in their basic components that, correspondingly, leads to high compressive strength. As the proportion of SSRS replacing cement increases, the lack of colloids in the CLSM internal pores and the fragility of the C-H-S gel during matrix formation lead to a decrease in pulse velocity, indicating lower compressive strength. Le and Nguyen [97] also carried out shear tests on stainless steel slag CLSMs. They demonstrated that cohesion developed with cement hydration, which was conducive to the improvement of shear strength. On days 7 and 28, a high amount of SSRS resulted in a greater decline in cohesion. This trend was consistent with that of unconfined compressive strength, which was due to SSRS contributing less significantly to strength compared with cement.

In addition to steel slag as an SCM, some studies have also reported that steel slag has been added into CLSM as an aggregate. Dinh et al. [99] and Y. Kim et al. [89] used original steel slag (OSS) and ground steel slag (GSS), respectively, as aggregates instead of natural sand to prepare CLSMs for geothermal heating and pump solutions. These CLSMs can be used as heat-resistant grouting. The results of their experiments showed that the performance of their CLSMs slightly decreased as the amount of steel slag that was substituted for natural sand increased. This was because the water absorption rate of the slag was higher than that of natural sand. The thermal conductivity of their CLSMs decreased with increasing OSS content but increased with increasing GSS content. This was because the intrinsic voids in the OSS particles increased with increasing OSS content, which enhanced the pore volume increase and reduced the thermal conductivity. In contrast, the GSS grinding process caused the steel slag particles to break, resulting in a reduction in the void volume. The GGS powder, therefore, filled the voids in the CLSM mixture after grinding, resulting in an increase in thermal conductivity. In addition, grinding contributed to pozzolanic reactions. This led to the generation of gels (C-S-H) that increased compressive strength and also filled pores within the mixture, further improving thermal conductivity. The strengths of these two slag CLSMs were both greater than that of a CLSM sample produced without replacing natural sand. This strength enhancement was due to the pozzolanic reactions of steel slag and fly ash. The strength of the CLSM with GSS replacing 30% of the natural sand was 2 times greater than the original natural sand CLSM. A higher natural sand replacement rate using GSS resulted in greater compressive strength. In contrast, although OSS was more beneficial for strength development compared to natural sand, the CLSM strength gradually decreased with an increasing replacement rate using OSS. These results demonstrate that the activity of steel slag increases after mechanical milling.

Due to the significant differences in the properties of steel slags obtained from different sources, it is recommended that sufficient laboratory research should be conducted before using steel slag CLSMs in engineering experiments to avoid potential adverse effects. This is true whether steel slag is added into CLSMs as a cementitious material or as an aggregate. The focus of research on the utilization of steel slag as a cementitious material should be on expansion damage. In particular, free calcium oxide and magnesium oxide cause a significant amount of expansion damage in CLSMs.

#### 2.2.4. Potential Application of Steel Slag Carbonization in CLSM

A review of the literature shows that the use of steel slag as an SCM for CLSM production has not been widely studied. This is mainly due to its characteristics: steel slag has a high free calcium and magnesium oxide content, low cementitious properties, and high heavy metal content. The high calcium content and high pH value (> 12) of steel slag mean that it reacts with CO_2_ to produce CaCO_3_ under appropriate conditions, making it a good material for CO_2_ mineralization and the production of clinker-free carbonate-bonded materials (CFCBM) [100]. Accelerated carbonization offers an effective solution to the problems of volume expansion, heavy metal leaching, and low cementitious properties caused by steel slag. Carbonizing steel slag is beneficial to the stability and activity of steel slag, as well as for carbon dioxide utilization. In the last five years, scholars have conducted a significant amount of research on steel slag carbonization. This research has focused on carbonization curing conditions, heavy metal leaching, and optimizing the carbonization process [101]. The CaCO_3_ produced by the reaction of calcium oxide, dicalcium silicate, and tricalcium silicate in steel slag with CO_2_ densifies the microstructure of the steel slag, reducing its pore size and total pore volume. This improves the compressive strength of the CSLM. In addition, CaCO_3_ particles produced by accelerated carbonization are relatively fine, which ensures nucleation points for hydration crystallization products and promotes the development of strength [102].

Zhong et al. [103] found that the compressive strength of a dry mixed block made from 100% steel slag powder (with a water–slag ratio of 0.12) was significantly enhanced to 69.9 MPa under a CO_2_ pressure of 0.55 MPa and a temperature of 70 °C for 360 min, while the compressive strengths of steel slag blocks produced without carbonization curing were only 4.4–7.5 MPa. This demonstrates that accelerated carbonized steel slag can be used as a binder for high-strength building materials. Mo et al. prepared a steel slag slurry by mixing 100% steel slag with water (with a water–slag ratio of 0.40). After curing the slurry for 14 days at a CO_2_ pressure of 0.1 MPa, its compressive strength reached 44.1 MPa [104]. The cementitious properties of CFCBMs that are produced with 100% steel slag are almost entirely dependent on the cementation ability of the calcium-rich carbonate, produced by accelerated carbonization. These cementitious properties are not dependent on the formation of any conventional adhesive, such as C-S-H [100]. Mo et al. [104] found that replacing 20% of the steel slag in the steel slag paste with cement significantly improved the compressive strength of the paste under CO_2_ curing. Ghouleh et al. [105] used an accelerated carbonization process to treat pressure-formed test blocks with a water–slag ratio of 0.40, and the strength of these blocks reached 80 MPa within 2 h and 109 MPa after 28 days. All of these studies investigated the accelerated carbonization of test blocks with a low water–slag ratio. Some other studies instead performed accelerated carbonization on steel slag grout with a high water–slag ratio before using it as an SCM to replace cement. For instance, Chen et al. [106] performed hypergravity carbonization on a grout with a water–basic oxygen furnace slag ratio of 20. This carbonized steel slag was used as an SCM to replace 10% of cement, and its usage reduced the setting time of cement and improved the cement’s early strength.

Yang et al. [107] prepared artificial steel slag aggregate by an accelerated carbonization method, using ethylenediamine tetra-acetic acid (EDTA) as a catalyst. Their experimental results showed that the concrete cast with steel slag catalyzed by EDTA had greater compressive strength and a more stable volume than the concrete cast with a steel slag aggregate that was not modified with EDTA. At present, no reports on the production of CLSM using carbonized steel slag have been retrieved. This includes the production of CLSM using accelerated carbonized steel slag as an aggregate, or the production of CLSM with undisturbed steel slag followed by performing accelerated carbonization treatment. Materials that undergo accelerated carbonization treatment are usually slurries with low water–slag ratios; this method obtains relatively high strength. Moreover, accelerated carbonization has become a novel and meaningful research topic for treating slurries with high water–slag ratios to obtain low strength (<8.3 MPa).

### 2.3. Red Mud

#### 2.3.1. Overview of Red Mud

**Figure 4:**
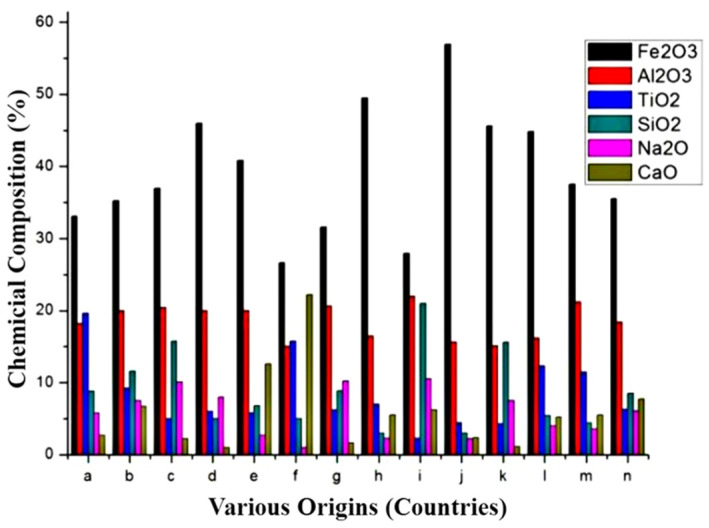
Chemical composition of red mud from various origins [108].

Bauxite residue, also known as red mud, is a solid waste obtained from alumina production. Red mud is an alkaline waste with a pH value of 10–13.5, and it contains a high concentration of heavy metals. Consequently, it is difficult to comprehensively utilize. It is estimated that the global alumina industry generates 7000 tons of red mud annually [108]. The content of each chemical component and the overall mineral composition of red mud can significantly vary, based on its source, technological process, and bauxite storage life (Figure 4) [108]. The processes used to refine alumina include the Bayer process and the combustion process. Alumina and iron oxide are the main components of red mud, which is generated by the Bayer process. This red mud contains fewer pozzolanic minerals due to its high iron, aluminum boehmite, and gibbsite bauxite content. Therefore, it is typically not used directly for building materials [108]. However, silicon oxide and calcium oxide are the main components of red mud that is generated by the combustion sintering process. These components are conducive to the direct use of this red mud as a building material. Currently, the Bayer method is the most widely used alumina-refining method. Some studies have used calcination, grinding, and other methods to treat Bayer red mud, resulting in high pozzolanic activity. The high alkalinity of red mud can also be used as an alkali source for alkali-activated binding materials. X. Liu et al. [109] reported the cementitious activity of red mud after high-temperature calcination. Their study showed that poorly crystallized Ca_2_SiO_4_ was generated after calcination at 600 °C, demonstrating the good cementitious activity of red mud.

#### 2.3.2. Performance and Mechanism of Red Mud CLSMs

Do and Kim [110] conducted research on the use of red mud as an alkali source for SCMs or as an alkali excitation material in CLSMs. They investigated the effects of partially replacing cement, fine aggregates, fly ash, or gypsum with red mud on the performance of CLSMs. Red mud was used as an SCM to partially replace cement and produce a CLSM. With pond ash as the aggregate, and cement and fly ash as the binder, they investigated the replacement of 0, 5, 10, 15, 20, 25, and 30% of the Portland cement with red mud, according to weight. Their findings demonstrated that the increasing red mud content slightly reduced the fluidity of their CLSM mixture. This was mainly because the specific surface area (SSA) of red mud is nearly 13 times larger than that of Portland cement. With increasing red mud content, the SSA of the solid mixture increased and the enhanced adsorption led to lower CLSM fluidity. This experiment adopted a fixed mix ratio, and the water consumption of all the CLSM mixtures was fixed. The enhanced adsorption was conducive to decreasing the bleeding water of the mixture and increasing its volume stability. Moreover, the plastic properties of red mud led to the retention of excess capillary water in the red mud-containing mixtures, another factor that influenced the reduction of bleeding in these mixtures. When the replacement amount of red mud was increased from 0% to 5%, the initial setting time of the mixture decreased and the initial setting time was slightly delayed. This was due to two main reasons. First, red mud contains a significant amount of Al_2_O_3_ (19.87%) and Na_2_O (14.92%). When mixed with water, red mud releases OH^−^, resulting in a highly alkaline environment, conducive to the hydration of Portland cement [111]. Second, the water consumption of the mixture was fixed in this experiment. Higher levels of red mud meant that the water used for cement particle bonding and hydration was more rapidly reduced and consumed, accelerating the initial setting process. The compressive strength of the CLSMs after 28 days of curing first increased and then decreased with the increasing red mud replacement rate. Replacing 15% of the cement with red mud resulted in the highest compressive strength of the corresponding CLSM (0.84 MPa). There were three main reasons for this strength development trend. First, the levels of Al_2_O_3_ (19.87%), Na_2_O (14.92%), and CaO (7.1%) were relatively high. When mixed with water, the calcium reacted with the alumina to form calcium aluminate. Second, the OH^−^ ions released by the red mud hydrolyzed the surface of the fly ash and pond ash, promoting the pozzolanic reaction of both ashes. Third, Si, Al, and Ca formed C-S-H gel through a dissolution reaction, improving compressive strength [112]. When more than 15% of the cement was replaced with red mud, the cement content was insufficient for developing strength. Moreover, the contribution of red mud to strength development was not high enough to overcome the decline in strength caused by the reduction in cement content.

### 2.4. Copper Slag

#### 2.4.1. Overview of Copper Slag

Copper dross is a granular technical waste obtained via copper smelting processes, and it can be used as a commercial material after grinding. Using copper slag as an aggregate instead of natural sand has no appreciable influence on the compressive force of concrete and can reduce its bleeding and shrinkage rates. In recent years, researchers have conducted studies on the properties of cementing materials with copper slag, which has shown great application potential as an SCM. However, it should be noted that the application of copper slag may be a radiation hazard and may lead to an elevated leaching risk of Cu, Pb, Zn, Ni, As, and other heavy metals [113]. After grinding copper slag, Junwei et al. [114] achieved pozzolanic activity stronger than that of fly ash. Their copper slag reacted with Ca(OH)_2_ to obtain C-S-H gel and improve the mechanical resistance of concrete.

#### 2.4.2. Properties and Mechanism of Copper Slag CLSMs

Taha et al. [115] completely replaced cement with copper slag to produce a CLSM. The 56-day compressive strength of this CLSM was very low, only 104–217 kPa. This strategy did not lead to a copper slag pozzolanic reaction in an alkaline environment without cement hydration. Hence, it is suggested that copper slag should be used in combination with cement to improve its pozzolanic activity. Copper slag has conductive characteristics because the content of Fe_2_O_3_ in copper slag is very high (64.6% by weight). Lim et al. [116] utilized copper slag and high-carbon fly ash to prepare a conductive CLSM. Increasing the amount of copper slag as an aggregate, replacing the sand, slightly increased the flow performance of the sample from 223 mm to 227 mm. Furthermore, the volume density increased after 28 days of curing, the compressive strength slightly decreased, and the conductivity increased. When copper slag was used to replace the fly ash, the tensile strength of the CLSM was enhanced. However, the cementing properties of copper slag were not discussed in this paper, and no data were provided showing that copper slag was added as an auxiliary cementing material.

**Figure 5:**
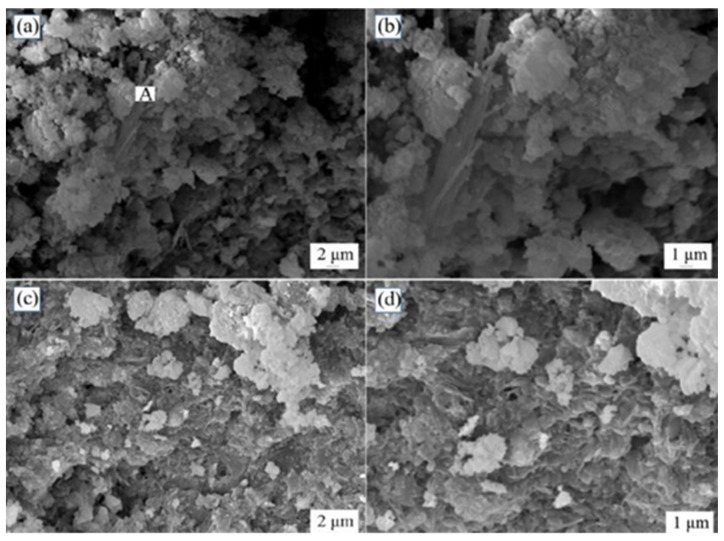
SEM microstructures of copper slag cementitious material after curing and hydration for (**a**,**b**) 7 days and (**c**,**d**) 28 days [30].

**Figure 6:**
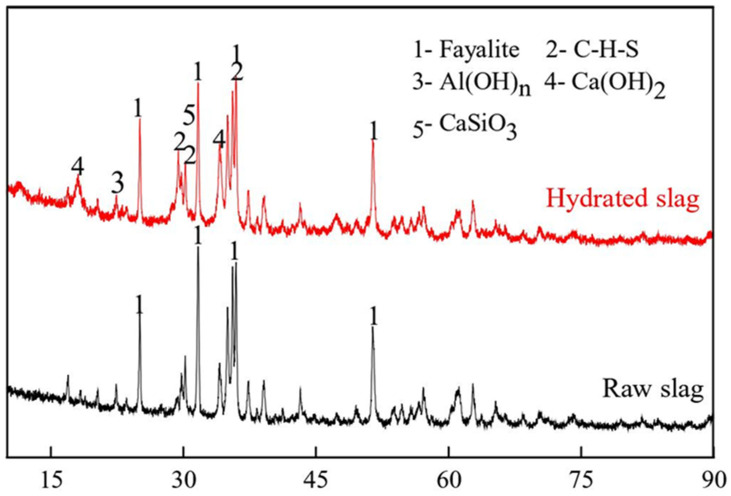
XRD patterns of original copper slag and hydrated sample products after 28 days [30].

According to the results of Taha et al. [115] and Lim et al. [116], the activity of copper slag is very low, and it is difficult to produce CLSMs using untreated copper slag SCMs. Even under the action of cement, copper slag has weak gelation properties and only makes a limited contribution to strength development. Lan et al. [30] used mechanical activation and soda stimulation to activate copper slag, producing a CLSM that met specific strength requirements. For instance, the particle size of copper slag can be reduced, its specific surface area can be enhanced, crystal defects can be induced, and the relative crystallinity of copper slag can be reduced during grinding. After milling for 80 min, the maximum SSA of the copper slag reported by Lan et al. reached 605 m^2^/kg, but an agglomeration phenomenon was evident between the copper slag particles. In addition, larger specific surface areas of the copper slag resulted in a higher grinding cost and lower production efficiency. Their experimental results showed that when the SSA of copper slag was greater than 520 m^2^/kg, the gelling strength caused by mechanical activation slowly increased with the increasing specific surface area. Therefore, it is recommended to grind copper slag for 60 min until its specific surface area is 520 m^2^/kg. Using lime, sodium hydroxide, and triethanolamine as chemical activators, an orthogonal experimental design was used. The experimental findings showed that the optimal amounts of lime, sodium hydroxide and triethanolamine were 28%, 2%, and 0.1%. Due to the use of this alkali activator, the crystal lattice of the slag was unevenly fractured and the glassy phase in the copper slag was dissociated. In Figure 5, images (a) and (b) show SEM images of a copper slag CLSM sample after curing and hydration for 7 days, and (c) and (d) show SEM images of the sample after curing for 28 days. These SEM images demonstrate that the active materials of the copper slag were constantly hydrated and that the hydration products were constantly increasing. Figure 6 shows a comparison of the XRD pattern of the sample when hydrated for 28 days with that of the original copper slag. After curing for 28 days, C-S-H, Ca(OH)_2_, and Al(OH)_n_ were present in the hydration products but no ettringite was produced. These results demonstrate that after mechanical activation and alkali activation, copper slag can completely replace cement. Meanwhile, the original copper slag can be used as an aggregate to prepare the CLSMs as mining backfill.

### 2.5. Other Smelting Waste

#### 2.5.1. Air-Cooled Blast-Furnace Slag

Air-cooled blast-furnace slag (ACBFS) is usually used as an aggregate in concrete because it has little or no binding ability due to its slow cooling process. ACBFS is also used as an aggregate to replace natural sand in the production of CLSMs. W.T. Lin et al. [32] reported a combination of water-quenched blast-furnace slag, cement, and circulating fluidized bed ash as cementing materials. Due to its high activity, the water-quenched blast-furnace slag was used as the main binder, with a dosage of 210 kg/m^3^. The dosage of cement was 60 kg/m^3^ and the dosage of the circulating fluidized bed ash was 30 kg/m^3^. A CLSM was produced by using circulating fluidized earth ash and ACBFS as aggregates. The results of a fluidity test, 24-hour drop-ball test, and compressive strength test were in accordance with the CLSM requirements for road backfilling and provided a basis for the resource utilization of both water-quenched slag and ACBFS. In order to broaden the application range of ACBFS, some scholars have investigated strategies for activating ACBFS. For instance, Matos et al. [117] attempted to grind ACBFS to enhance its activity and used it as an SCM to produce self-compacted concrete. The addition of finely ground particles provided an additional surface area for the formation and growth of hydrochemical products. Thus, the hydration kinetics of the cement were enhanced. These results show that ACBFS can be used as an SCM in the production of self-compacted concrete, and the overall performance of ACBFS is equivalent to that of limestone filler. Hence, prior to large-scale use, additional tests such as durability surveys are recommended.

#### 2.5.2. Jarosite Residue

Jarosite residue (JR) is a smelting waste with little commercial value, derived from the zinc industry, so the disposal of JR is a major sustainability concern for zinc factories. Bouzalakos et al. [118] and Chan et al. [119] attempted to use JR for CLSM production, but no pozzolanic hydration products of JR were found in these studies. When JR was used to partially replace silica sand for CLSM production, the compressive strength of the CLSMs was reduced and the leached lead and zinc content was many times higher than guide values. Therefore, raw JR is not recommended for use in CLSM production or as an SCM.

#### 2.5.3. Ferrochrome Slag

Ferrochrome slag (FCS) is the waste slag generated by the production of ferrochrome alloys. The annual global production of ferrochrome slag is about 10 million tons, and production is increasing at a rate of 2.8–3.6% per year [120]. The SiO_2_ content in FCS accounts for 21.28–35.60% of its total mass, while MgO usually accounts for 20.85–38.5%, and Al_2_O_3_ accounts for 20–30%. FCS also contains CaO, Cr_2_O_3_, Fe_2_O_3_/FeO, and other chemical compounds. The total SiO_2_, Al_2_O_3_, and Fe_2_O_3_ contents of FCS account for over 50% of its entire volume, indicating that FCS may have some pozzolanic properties [121]. Similar to blast-furnace slag, the cooling process of liquid FCS has a significant impact on its crystallinity and particle size. Usually, rapid water-cooling produces small (5 mm) dark grains with a solid rock structure, while air-cooled ferrochrome slag is typically larger (8 to 20 mm) and consists of lumpy-grained gray particles. The crystallinity of air-cooled FCS is significantly higher than that of water-cooled FCS, with a mainly crystalline structure and negligible glass phase content [122]. Air cooling is a common curing process for liquid FCS. At present, there is little research on the use of FCS as a building material, and FCS has not been widely exploited as a useful product. However, FCS has greater crushing resistance, wear resistance, and impact resistance compared with traditional limestone, a rough and irregular surface, and a large number of stable crystalline phases [121]. Thus, most existing research on the use of FCS as a building material has focused on its use as a natural sand replacement to produce concrete. Kauppi and Pekka [123] showed that the mechanical and physical properties of air-cooled FCS were better than those of various natural aggregates. At present, only a few studies have focused on the cementitious properties of FCS. For example, Nath [122] used an alkaline solution mixed with sodium hydroxide to excite FCS for the preparation of polymers. Zhou et al. [124] used a compound chemical activator composed of NaCl, Na_2_SO_4_, NaF, and Al_2_(SO_4_)_3_ to effectively activate low-carbon chromite slag. Işil and Hasan [125] evaluated the potential of FCS as an SCM for preparing mortar, and their SEM and XRD results showed that FCS has pozzolanic activity and that it shows potential as a cement-based SCM.

Only one reference was retrieved on the incorporation of FCS into CLSMs. Mahamaya and Das [28] incorporated FCS into a CLSM as an aggregate or cementing material. XRD analysis showed the presence of an aluminosilicate glass phase in their FCS pellets. Moreover, Al, Si, Ca, and Mg were observed on the surface of the FCS pellets under an optical microscope, which is helpful for the pozzolanic reaction. The FCS particles in this work were irregular in shape, slender, and had a smooth surface. This research showed that the compressive strength of the resulting CLSM decreased when FCS was used as an SCM instead of fly ash, indicating that the pozzolanic activity of FCS is lower than that of fly ash. After 56 days of solidification, the minimum uniaxial compressive strength (UCS) of a CLSM prepared by FCS as the aggregate or cementing material was 3.79 MPa, and the maximum UCS value increased to 18.32 MPa. Therefore, the developed CLSM was suitable for use as a structural backfill and in trenchless backfill projects. It is worth pointing out that CLSM strength can also be lowered by reducing the amount of cementing materials, such as cement, so that the compressive strength meets the maximum strength limit required for backfill materials. It should also be noted that chromium is a harmful mineral, especially hexavalent chromium. Hence, experiments should be carried out to ensure that Cr leaching meets the requirements of environmental authorities before recycling FCS for use in these applications.

### 2.6. Summary

To facilitate a comparison of the characteristics of various types of metallurgical slag and their effects on the properties of CLSMs, several typical metallurgical slag types are summarized in Table 3. Under normal circumstances, the activity of untreated metallurgical slag is low, due to its large size. Therefore, untreated slag is not typically used in its initial form. After grinding treatment, the surface area in contact with water or other solutions is enhanced, which increases the activity. In general, the activities of water-quenched blast-furnace slag and steel slag are higher than that of other metallurgical waste slags. In particular, the activity of steel slag is rapidly stimulated under an accelerated carbonization treatment. As well as grinding, other metallurgical waste slags can be used in combination with cement, quicklime, and other cementing materials to further enhance activity.

## 3. Cement-Free CLSMs

The most important factor affecting the price of CLSMs is the cost of cement as a raw material. Typical CLSM blends require 5–10% cement, while conventional CLSM fillings require unconfined compressive strengths of less than 1.0 MPa and only 3–7% cement by weight when re-excavated. As an important raw material for CLSM strength control, cement was once considered an indispensable component of CLSMs. Compared to concrete, CLSMs have a low cement level, but the overall cement consumption used for the mass production of CLSMs is still high [126]. Therefore, with the goal of cost reduction, scholars have explored reducing cement or not using cement at all to produce CLSMs. The research summarized in Section 2 shows that good results have been obtained in CLSM production by partially substituting cement with one or more auxiliary cementing materials, and some of this research has been successfully applied in practical projects.

For economic and sustainable development reasons, the production of CLSMs without cement has become a popular trend because the production of Portland cement requires the consumption of limestone and clay, uses a significant amount of energy, and leads to high carbon dioxide emissions. Therefore, cement-free clinker concrete materials containing metallurgical waste slag have been extensively studied. Cui et al. [127] used GBFS, steel slag, and desulfurization gypsum as cementing materials to prepare a clinker-free concrete. Ettringite (AFt) and C-S-H gels were formed through the synergistic effect of these solid waste products. D. Xu et al. [128] prepared clinker-free concrete by combining ammonia–soda residue (ASR), with various metallurgical slags as cementing materials and iron tailings as an aggregate. The ASR and its leachate were strongly alkaline. Together with steel slag, they offered a sufficiently alkaline environment for reactions in granulated blast-furnace slag. Therefore, slag hydration generated silicate and aluminate compounds, while the chloride and carbonate in the ASR accelerated these reactions. In Korea, a cementless binder (CB) made from GBFS and other alkali activators has emerged as a cementing material for CLSM production. An increasingly wide range of cement substitute materials and cement-free CLSMs have been investigated by scholars.

Current CBs for CLSMs typically contain self-gelling by-products and alkali-excited by-products. Self-gelling by-products are used to generate the desired CLSM strength without using cement or other activators. For example, Lim et al. [129] used high-calcium fly ash as the only binder to prepare a CLSM that met specified performance requirements. SEM, EDS, and XRD analysis of this hardened CLSM showed that the high-calcium fly ash underwent geological polymerization to produce aluminum calcium silicate hydrate (C-A-S-H), and underwent hydration to produce hydrated calcium sulfoaluminate. These processes provided the CLSM with the required strength. L. Du et al. [130] prepared a quick-setting CLSM for abutment backfilling, using high-calcium fly ash, natural sand, and water. The high calcium oxide (CaO) content was the main factor behind the early condensation and greater early strength of this CLSM. Due to its rapid solidification and hardening behavior, the early formation of ettringite consumed a large amount of the available water, so no bleeding was observed. With high-calcium fly ash as the only binder, the ratio of water to fly ash affected the hydration and hardening of the binder. A higher ratio of water to fly ash meant lower strength at different aging times. These studies demonstrate the preparation of CLSMs by using self-cementing by-products. However, there are only a few studies that investigate this topic, and more scholars have used alkali excitation by-products to prepare cement-free CLSMs.

The sustainability of CLSMs is enhanced by eliminating cement and using activated by-products as the sole binder. Alkali-activated binding materials (AAMs) can be used as the binders in these cement-free CLSMs. In AAMs, aluminosilicate materials are activated by a high alkalinity solution. Activated calcium, silicate, and alumina in the precursors are rapidly dissolved under the highly alkaline conditions and are released into the solution. Subsequently, polymerization occurs to form C-S-H, C-A-S-H, and sodium aluminum silicate hydrate (N-A-S-H) gels. This enhances the performance of the CLSMs with AAMs [131]. Compared with common cement, AAMs have excellent performance and show good potential for replacing cement in CLSMs. In existing studies, the reported metallurgical slag materials that can completely replace cement in CLSMs mainly include: slag activated by alkaline chemical solutions, such as NaOH, Ca(OH)_2_, and sodium carbonate; alkaline by-product activated slag, such as phosphogypsum, waste lime, and cement kiln ash [83]; slag activated by alkaline substances in the regenerated aggregate; and high-calcium fly ash activated slag [129].

### 3.1. Alkaline Chemical Solution-Activated Slag

#### 3.1.1. Liquidity

Chemical reagents, such as NaOH, KOH, sodium carbonate, and sodium silicate, are often used as alkali activators, but these chemical reagents are commodities with high unit prices. The majority of studies on the preparation of CLSMs from slag activated by alkaline chemical solutions use a mixture of NaOH and distilled water as the alkaline activator, and these studies use this solution to simultaneously activate slag and fly ash. N.K. Lee et al. [132] prepared a cement-free CLSM by using a NaOH solution as the alkaline activator, with fly ash and GBFS as the binder, and bottom ash as a fine aggregate to replace natural sand. Their results showed that the dosage of solid NaOH and GBFS did not significantly influence the fluidity of the CLSM mixture. The main factors influencing fluidity were the water–binder ratio and the ratio of the bottom ash. S.M. Park et al. [133] showed that with fixed water content, increasing the content of NaOH reduced the fluidity of the fresh CLSM. With increasing slag content, the hydration degree of the CLSM was enhanced and more mixing water was consumed. Thus, the bleeding rate decreased. Increasing the NaOH content accelerated the activation reaction, reduced the condensation time, and slightly reduced the bleeding rate.

#### 3.1.2. Compressive Strength and Its Mechanism

The compressive strengths of alkali-activated CLSMs are related to many factors, such as the amount of slag and fly ash, the molar concentration of NaOH solution, and the ratio of water to the binder. The addition of the NaOH solution chemically activates the fly ash and slag, resulting in the formation of C-A-H and C-A-S-H. Research shows that the compressive strength of fly ash polymers increases with increasing slag volume [134]. Similarly, the compressive strength of CLSMs increases with increasing GBFS volume. This is because the addition of more slag further promotes hydration and increases the amount of C-S-H gel. In turn, this leads to a hardened and dense matrix, as well as significantly enhanced compressive strength. The results of S.M. Park et al. [133] also confirm this view. Ghanad and Soliman [135] believe that alkali-activated cementless CLSMs show greater compressive strength compared with traditional cement-based CLSMs. This is because when a NaOH solution is used to activate fly ash and slag at ambient temperatures, Ca^2+^, Si^4+^, and Al^3+^ ions are released, and a polymerization reaction occurs. The added GBFS reacts with the NaOH solution more quickly than fly ash. Due to the high calcium content of slag, a hydration reaction occurs in addition to geological polymerization, and greater strength can be obtained under this dual reaction [136].

Increasing the content of the alkali activator NaOH also promotes the development of cementless CLSM strength. This is because the presence of more OH^−^ ions promotes the removal of silicon, aluminum, and calcium from the slag, which leads to the formation of more C-S-H gels through polymerization. Meanwhile, OH^−^ ions increase the degree of slag hydration, leading to a significant in the compressive strength of CLSMs. In particular, the early compressive strength of these CLSMs significantly increases with increasing NaOH dosage, which is very beneficial for emergency repair projects. The particle size of GGBFS is very small, with an average particle size of only 14.5 μm. Therefore, the alkali activation reaction rate of GGBFS is faster than that of fly ash, and the addition of GGBFS can also lead to an enhanced early strength in CLSMs. However, the effect of GGBFS on the reaction rate is lower than that of NaOH. Therefore, NaOH plays an instrumental role in enhancing the early strength of cementless CLSMs, while GGBFS improves the longer-term strength of cementless CLSMs [74].

Sodium carbonate and sodium silicate are also basic activators that can be used instead of NaOH. For example, Kim et al. [126] used sodium silicate to stimulate blast-furnace slag as a cementless binder, to produce a CLSM. It should be noted that in cementless CLSMs that are produced with alkaline chemical solution-activated slag as a binder, these chemical activators are major factors in controlling the cost of CLSM.

### 3.2. Alkaline By-Product Activated Pozzolanic Waste

#### 3.2.1. Phosphogypsum and Waste Lime-Activated Slag

In recent years, a great deal of research has been performed on the preparation of hemihydrate gypsum from phosphogypsum, or the synergistic effect of phosphogypsum with other materials as cementing materials [137,138]. Ting et al. [139] found that slaked lime improved the mechanical strength and water resistance of phosphogypsum slurry. Lu et al. [140] converted phosphogypsum into hemihydrate gypsum and mixed it with fly ash and cement, to obtain a new type of waterproof cementing material. The dosage of hemihydrate gypsum in this cementing material reached 70%. Harrou et al. [141] added steel slag and phosphogypsum to a mixture of bentonite and lime to accelerate a hydration dynamic reaction. The generated C-S-H and ettringite improved the strength of the mixture. The maximum compressive strength of this mixture reached 1.91 MPa, showing the feasibility of its use as a roadbed material with low strength requirements. Other reported results have shown that hemihydrate phosphogypsum has strong cementitious properties under certain activation conditions and can be used to produce low-strength backfill materials [142,143]. Rong et al. [144] formulated a cementing material with hemihydrate phosphogypsum and a small amount of lime; this cementing material completely replaced cement as a filling material for goaf areas. The strength of this filling body reached the expected target strength of 2.5 MPa within 3 days. Moreover, it displayed the advantages of controlled setting time, high early strength, a low bleeding rate, and good fluidity.

To reduce the cost of producing CLSMs from alkali-activated by-products and improve the utilization rate of solid waste, the use of alkaline by-products such as gypsum, red mud, and cement kiln ash as activators has become a research hotspot. Do et al. [145] produced a cementless binder (CB) by adding phosphogypsum and waste lime to GBFS. They then prepared a CLSM, with fly ash and pond ash as aggregates. Their results showed that the fluidity of their cementless CLSM was mainly controlled by the addition of water to the mixture. Increasing the water–binder ratio or water–solid ratio improved the flow performance of the CLSM but also enhanced the bleeding rate. Due to the existence of GBFS in this CB, the fluidity of the CLSM prepared using the CB was slightly higher than that prepared with the same amount of cement. However, the bleeding rate of the CLSM prepared with the CB was high [145,146]. Compared with the cement-based CLSM, the CB-based CLSM required a longer setting time due to the presence of GBFS, which is a weak hydraulic material. When the amount of water was fixed, the fluidity of the CLSM increased with increasing water–CB ratio, while the compressive strength decreased, due to the lower amount of CB.

Do et al. [145] compared the influence of CB and cement on the hardening properties of CLSMs. At each curing stage, the compressive strengths of all their CB-based CLSMs were slightly higher than those of cement-based CLSMs. When the CB was mixed with water, OH^−^ ions were released that enhanced the alkaline environment of the solution. These OH^−^ ions hydrolyzed the surface of the pond ash or fly ash to release Si and Al ions, and they reacted with the Ca ions produced by the lime to form C-S-H gel [132]. Moreover, the phosphogypsum in the CB led to the reaction of (SO_3_)_2_^−^ ions with Al^3+^ released from the GBFS and fly ash, as well as with Ca^2+^ ions generated by lime hydration. Thus, ettringite was formed. With an appropriate gypsum content, the excess Ca^2+^ and Al^3+^ ions also reacted to form C_4_AH_13_ [147]. XRD and SEM analyses confirmed that the CLSM produced with CB generated C-S-H, ettringite, and C_4_AH_13_. The gel and ettringite filled the pores of the sample and hardened the CLSM mixture, resulting in enhanced strength. XRD and SEM analyses of the CLSM that contained only cement showed that C-S-H and calcium hydroxide were generated. Therefore, the unconfined compressive strength of the CB-based CLSM was slightly higher than that of the cement-based CLSM.

Do et al. [145] showed that both the cement-free CLSM and cement-containing CLSM solutions were alkaline but that the pH values of the cement-free CLSMs were lower than those of the cement-containing CLSMs, with correspondingly lower corrosivity. Do et al. [148] also reported the effects of curing conditions on the hardening performance of cement-free CLSMs. Their research showed that the 28-day compressive strength of a CLSM mixture under wet conditions was significantly higher than that under saturated curing conditions. During saturated curing, excess water enters the pores of the specimen and hinders the hydration reaction of the cementitious material. Furthermore, the thermal conductivity of the CB-based CLSM was satisfactory compared with that of traditional grout. Under SC conditions, the heat resistance of the cement-free CLSM was significantly higher than that produced using wet-room curing. A high thermal conductivity is beneficial for cable backfilling, which may generate heat, and CLSMs with high heat resistance can be used as an alternative grout material for borehole heat exchangers in ground source heat-pump systems.

Y. Kim et al. [146,149] used gypsum and lime-activated slag as cementing materials and excavated soil to partially replace fly ash as an aggregate. When the proportion of excavated soil to fly ash was increased, the CLSM fluidity decreased, the bleeding rate decreased, and the compressive strength decreased. The compressive strength of the CLSMs that were produced with more excavated soil was lower because fly ash has pozzolanic activity, while excavated soil does not. Increasing the proportion of soil resulted in a reduction in fly ash consumption. Consequently, the contribution of fly ash, when excited by lime, to CLSM strength development was also reduced.

#### 3.2.2. Cement Kiln Dust-Activated Slag

The chemical composition of cement kiln dust (CKD) is similar to that of cement, but CKD has a higher alkali content. Therefore, CKD can be used as an alkaline activator. Lachemi [83] prepared cementless CLSM using CKD-activated slag. With a fixed CKD dosage, the addition of GBFS did not significantly influence the workability of the CLSM. However, the CLSM strength increased. In particular, the strength of the CLSMs was significantly enhanced when the GBFS dosage was higher than 50 kg/m^3^. In addition to this positive effect, reactions between the GBFS and CKD also contributed to the strength development of this CLSM.

#### 3.2.3. Red Mud as an Alkaline Activator

According to the research of Do and Kim [110], red mud only provides a weak pozzolanic activity. Before the pozzolanic activation reaction, the main roles of red mud are to provide OH^−^ and to increase the alkalinity of the CLSM mixture, which enhances the reactions of pozzolanic materials. Therefore, some scholars have attempted to use red mud as an alkali source to reduce the cost of alkali-activated cementing materials. Yuan et al. [29] used fly ash and cement as binders and a varying amount of red mud to replace fly ash. The pH value of their solution increased at all aging durations with increasing red mud content. This was due to the direct dissolution of NaOH in the red mud and the calcium hydroxide formed by the Na_2_CO_3_ in the red mud and cement. The high pH value promoted the dissolution rate of silica and alumina from the fly ash and accelerated the hydration of the cement and ash. Thus, a high level of polycondensation and better strength development were achieved. Replacing the ash enhanced the compressive strength of the CLSMs after all aging periods, and the compressive strength of these CLSMs was enhanced with increasing red mud content. This trend differs compared with that reported for CLSMs that are produced by using red mud to replace cement, in which the strength first increased and then decreased with increasing red mud content [110]. Increasing the red mud content up to 40 wt % reduced the porosity of the CLSM. However, raising the red mud content to 60 wt % enhanced the porosity of the CLSM. Although the CLSM containing 60% red mud had the highest porosity, it also had the lowest number of harmful pores (≥50 nm). Therefore, red mud contributes to the microstructure densification and strength development of CLSMs via two main mechanisms. The addition of red mud chemically accelerates the reactions of cementing materials and physically densifies the microstructure of hardened CLSMs.

Do et al. [150] produced cementitious BC using fly ash, red mud, lime, and phosphogypsum to completely replace the cement in the CLSM. The dosages of fly ash and lime in the cement-free BC were fixed, and the dosages of red mud and phosphogypsum varied, as described by the ratio of phosphogypsum to red mud (G/Rm). A higher G/Rm value potentially resulted in decreased CLSM fluidity, bleeding, sedimentation, and early strength. However, the 28-day strengths of CLSMs with higher G/Rm ratios were enhanced. Their results showed that the red mud and phosphogypsum in the newly developed binder potentially affected the early strength and long-term strength of the mixture, respectively. Another study by Do et al. [151] also proved that an enhanced G/Rm ratio potentially led to decreased fluidity and prolonged the initial setting time.

Do et al. [152] prepared a slag incorporated in an inorganic binder as a cement substitute, but the compressive strength of the CLSM, prepared with this CB and pond ash, was less than 0.51 MPa, slightly below the required strength. These results show that mixing red mud and pond ash to produce an artificial aggregate can improve the engineering performance of a pond-ash-based CLSM when bonded with non-cementing materials. This experiment showed that the main reason for the improved resistance of the CLSM was the good pore-filling performance and the high Na_2_O content of the red mud artificial aggregate, which stimulated the pozzolanic reaction.

### 3.3. Recycled Aggregate-Activated By-Products

The residual slurry on the surface of recycled aggregate (RCA) contains alkali compounds, the cement hydration product, calcium hydroxide, and unhydrated cement particles. When mixed with water, the alkali and calcium hydroxide dissolve, while the unhydrated cement particles hydrate to form calcium hydroxide. This makes the solution alkaline and stimulates the activity of pozzolana metallurgical waste residue. Achtemichuk et al. [153] used RCA to stimulate GBFS and high-calcium fly ash (HCFA) to prepare cement-free CLSMs. The compressive strength of their CLSMs increased with an increasing GBFS or HCFA incorporation ratio. Because the content of calcium oxide in the GBFS was significantly higher than that in the HCFA, the compressive strength of the CLSM containing GBFS was greater than that of the CLSM with the same HCFA dosage. The strength of this cement-free CLSM was mainly due to three reasons. The first reason was the hydration reaction of unhydrated cement particles in the RCA. However, this effect did not significantly contribute to the strength of the CLSM. The second reason was the hydration of slag and HCFA, due to their self-cementitious properties. The third reason was the pozzolanic reaction between the slag and HCFA, stimulated by the alkaline solution, mixed with RCA and water. Furthermore, the Ca(OH)_2_ of the RCA also promoted the pozzolanic reaction to a certain extent. With an increasing amount of slag, the cohesive properties of the mixture were enhanced, and CLSM bleeding was reduced, thus reducing the amount of sedimentation. When the RCA was used to replace partially recycled fine aggregate, the hardening time or time-to-loading of the cementless CLSM was shortened. This pozzolanic reaction, combined with the autohydration of slag and HCFA, is thought to contribute to the hardening and strength development of CLSMs.

## 4. Conclusions

Due to the low strength requirements of CLSMs, most relevant literature shows that a measure of low-quality metallurgical waste can be used as a cementing material or an aggregate in CLSM production. As a cementing material, metallurgical waste reduces the consumption of cement and provides a strategy for waste disposal, reducing the pollution threat that this waste can otherwise pose to the environment.

The activity of metallurgical waste slag is related to its physical and chemical properties, and it is also affected by other factors, such as pretreatment, environmental alkalinity, and curing conditions. Metallurgical waste slag is the most commonly used SCM because of its excellent physical and chemical properties. In addition to its good pozzolanic properties, red mud is also highly alkaline, so it can be used as an activator of alkali-activated materials. Generally, water-cooled slag contains a high glass phase proportion, due to the rapid cooling achieved by water-cooling processes, and its activity is higher than that of air-cooled slag.

The mix proportion is an important factor in the reaction of CLSMs, and water consumption has a significant influence on the flow performance of CLSMs. When the proportion of other materials is fixed, the fluidity of CLSMs increases with increasing water consumption, with correspondingly higher water–binder ratio and water–solid ratio. However, the excessive addition of water to enhance fluidity will also lead to bleeding, segregation, and the strength reduction of mixtures. The smooth surface of GGBFS can be used to help CLSMs achieve good fluidity. The compressive strength of CLSMs is mainly related to the dosage of cementing material or mixing water. A greater amount of cementing material (metallurgical slag + cement) means a higher compressive strength in the CLSM. However, the contribution of metallurgical slag SCMs to strength development is less than that of cement. Therefore, replacing cement with an increasing amount of SCM reduces compressive strength. Most SCMs tend to hydrate slowly, so the long-term compressive strength development of CLSMs tends to increase with increasing SCM content. Consequently, careful attention should be paid to CLSM selection for projects requiring excavation.

In general, the production of CLSMs with metallurgical slag as a cementing material is feasible in terms of engineering performance, cost, and environmental impact. In the future, there should be a greater research focus on other metallurgical slags besides mineral waste residue, and research on all solid-waste cementless CLSM cementing materials should also be strengthened. In order to study the strength development mechanisms of CLSMs that are prepared using metallurgical slag as an SCM, microscopic research methods, such as CT, XRD, SEM, and EDS, should be used to offer a rationale for the further resource utilization of metallurgical slag materials as SCMs.

## Figures and Tables

**Table 1:** Chemical ingredients of granulated blast-furnace slag and their sources (wt %).

Source	SiO_2_	Al_2_O_3_	Fe_2_O_3_	CaO	MgO	SO_3_	TiO_2_	MnO	Reference
Shougang Group	33.50	12.52	1.10	37.90	9.29	2.51	N/A *	N/A	[50]
Baosteel	33.54	14.83	1.20	40.06	8.43	0.12	0.60	0.43	[50]
Tangsteel	30.80	14.70	2.67	38.10	8.84	2.77	0.83	0.13	[50]
Datong	27.90	17.30	3.60	37.90	8.37	1.90	1.87	0.22	[50]
Handan	31.00	13.70	1.15	38.60	10.30	0.99	1.66	0.24	[50]
Chengdu	28.80	12.20	4.65	36.70	6.41	1.40	7.38	0.91	[50]
China	25.56	12.85	1.11	51.65	2.95	2.8	1.17	N/A	[51]
Poland	38.7	7.7	0.6	40.5	6.32	0.31	N/A	N/A	[52]
Nippon Steel	32.51	14.37	0.15	43.98	5.17	3.03	N/A	N/A	[53]
Pakistan	37.22	10.37	1.23	35.66	N/A	0.34	N/A	N/A	[54]
South Korea	29.13	11.82	0.44	42.51	2.43	3.34	0.59	0.23	[55]
Spain	35.96	10.61	0.4	42.89	7.10	2.02	N/A	N/A	[56]
India	36.9	14.1	0.11	40	8	N/A	N/A	N/A	[57]
Australia	34.5	14.5	N/A	40.5	6.5	N/A	1.5	0.5	[58]

* N/A: not available.

**Table 2:** Current typical slag standards in China, Japan, Korea, and the EU.

Standard/Specification		China GB/T 18046-2017 [61]			Japan JIS A 6206:2013 [62]				Korea KS F 2563 (2019 Confirm):2012 [63]			EU EN 15167 -1:2006 [64]
Grade		S105	S95	S75	8000	6000	4000	3000	Class 1	Class 2	Class 3	N/A *
Density/(g/cm^3^)		≥2.8			≥2.8				≥2.8			N/A
Specific surface area/(m^2^/kg)		≥500	≥400	≥300	700–1000	500–700	350–500	275–350	800–1000	600–800	400–600	≥275
Activity index (%)	7 d	≥95	≥70	≥55	≥95	≥75	≥55	N/A	≥95	≥75	≥55	≥45
	28 d	≥105	≥95	≥75	≥105	≥95	≥75	≥ 60	≥105	≥95	≥75	≥70
	91 d	N/A	N/A	N/A	≥105	≥105	≥95	≥80	≥105	≥105	≥95	N/A
SO_3_ (%)		≤4.0			≤4.0				≤4.0			≤2.5
MgO (%)		N/A			≤10				≤10			≤18
Glassiness (%)		≥85			N/A				N/A			N/A

* N/A: not available.

**Table 3:** Effects of metallurgical waste slags on CLSM performance.

Metallurgical Waste Slag	Main Components	Phase or Mineral	Particle Morphology	Cementitious Properties	Reaction Products	Application Mode	Setting Time	Workability	Strength	Reference
GGBFS	CaO, SiO_2_, Al_2_O_3_, MgO	Glassiness accounts for 80–90%	Smooth and compact surface	Weak hydration when <45 μm; pozzolanic activity	C-S-H	Replace cement	↑ ^1^	↑	↓ ^2^	[43,59,72,74]
Steel slag powder	CaO, SiO_2_, Al_2_O_3_, Fe_2_O_3_,	C_2_S, C_3_S, C_4_AF	N/A *	Weak hydration; pozzolanic activity	C-S-H, Ca(OH)_2_, Ettringite	Replace aggregate	↑	N/A	↑	[93,94]
Raw steelmaking slag	CaO, SiO_2_, Al_2_O_3_, Fe_2_O_3_,	N/A	N/A	Very weak hydration	C-S-H	Replace aggregate	↓↑ ^3^	↓	↑↓ ^4^	[89,99]
Ground steelmaking slag	CaO, SiO_2_, Al_2_O_3_, Fe_2_O_3_,	N/A	N/A	Weak hydration	C-S-H	Replace aggregate	↓	↓	↑	[89,99]
SSRS	CaO, SiO_2_	N/A	N/A	Weak hydration; pozzolanic activity	C-S-H	Replace cement	↑	↑	↓	[97,98]
Red mud	Fe_2_O_3_, Al_2_O_3_, SiO_2_, Na_2_O	N/A	N/A	Alkali stimulates hydration	Ca(OH)_2_, calcium Aluminates	Replace cement	↑↓	↓	↑↓	[110]
Copper slag	Fe_2_O_3_, SiO_2_	N/A	N/A	Weak pozzolanic activity	N/A	Replace cement	N/A	↓	↓	[115]
Copper slag	Fe_2_O_3_, SiO_2_	N/A	N/A	N/A	N/A	Replace aggregate	N/A	↑	↓	[116]
						Replace fly ash	N/A	↑	↑	
FCS	Al_2_O_3_, SiO_2_, MgO, Cr_2_O_3_	Presence of glass phase	Irregular shape, smooth surface	N/A	N/A	Replace 66% cement and fly ash	N/A	↓	↓	[28]
						Replace 55% cement and fly ash	N/A	↑	↓	

* N/A: not available. ^1^ ↑: Increase. ^2^ ↓: Decrease. ^3^ ↓↑ indicates a decrease and then an increase. ^4^ ↑↓ indicates an increase and then a decrease.

## Data Availability

Data are available in a publicly accessible repository.
